# A Bibliometric Analysis and Visualization of the Current Status and Future Perspectives of Adrenal Cortical Adenoma

**DOI:** 10.7759/cureus.92367

**Published:** 2025-09-15

**Authors:** Zhaoqi Liang, Shuying Wang, Xuan Lan, Zengzhao Wei, Encun Hou

**Affiliations:** 1 Graduate School, Guangxi University of Chinese Medicine, Nanning, CHN; 2 Oncology, Ruikang Hospital, Guangxi University of Chinese Medicine, Nanning, CHN

**Keywords:** adrenal cortical adenoma, bibliometrics, cancer, citespace, vosviewer

## Abstract

Adrenal cortical adenoma (ACA) is a tumor of the adrenal cortex with complex pathophysiological mechanisms often related to hormonal abnormalities such as aldosterone hypersecretion. This bibliometric overview of ACA research charts its growth, trends, and emerging themes. Publications on ACA were extracted from the Web of Science Core Collection until June 1, 2025. VOSviewer and CiteSpace software were used for co-occurrence, co-citation, and keyword burst analyses. We analyzed the temporal distribution and networks of countries, institutions, journals, authors, and keywords. A steady increase in the number of ACA publications was observed, with a peak from 2011 to 2015. The leading contributors were from the United States, Japan, and Italy, with significant contributions from institutions such as Tohoku University and the University of Würzburg. Co-citation analysis revealed a focus on histopathological classification, diagnosis, and the molecular mechanisms of ACA. The bibliometric analysis identified key trends in ACA research, such as the growing emphasis on genetic mechanisms and clinical guidelines. Interdisciplinary collaborations have enhanced the understanding of ACA.

## Introduction and background

Adrenal cortical adenoma (ACA) is associated with complex pathophysiological mechanisms. Mast cells play a role in aldosterone hypersecretion in aldosterone-producing adenoma (APA). Research has shown that the density of mast cells is higher in APA tissues than in normal adrenal tissues. These mast cells, primarily observed in the adrenal cortex adjacent to adenomas or within adenomas, can be divided into two groups. A subset of adenomas with a high density of intratumoral mast cells is correlated with aldosterone synthase expression and in vivo aldosterone secretory parameters. Conditioned medium from human mast cell line cultures can induce an increase in aldosterone synthase mRNA expression and aldosterone production in human adrenocortical cells [1].

The study of ACA has a long-standing history. The first autopsy case report of a child with what is likely adrenocortical carcinoma was described by Sampson in 1697. At that time, the adrenal gland was not generally known as a separate organ, and the report indicated that the disease arose from the left kidney, with no indication of adrenal origin. This early case report is instructive when comparing the medical knowledge of the 17th century with the current understanding [2].

The prevalence of ACA varies among different populations. Adrenal incidentalomas, often including adenomas, are found in approximately 4% of patients undergoing abdominal imaging, with a peak prevalence in the sixth and seventh decades of life [3]. In the context of Cushing’s syndrome, screening studies suggest that only 1% of hypertensive patients have secondary hypertension due to Cushing’s syndrome, and among patients with osteoporosis and vertebral fractures, up to 5% were diagnosed with subclinical hypercortisolism, most of whom had adrenal adenomas [4].

In recent years, with the rapid development of molecular biology techniques, endoscopic diagnosis and treatment, immunotherapy, and other advancements, research on ACA has gradually increased; however, bibliometric analyses on this subject have not been undertaken. Consequently, this study offers an exhaustive overview of the knowledge framework and research trajectories in this area by employing bibliometric analysis.

## Review

Methodology

Search Database

The Web of Science Core Collection (WoSCC) is a widely acknowledged, leading, and exhaustive database for scientific research, spanning a broad spectrum of academic publications and investigations. The WoSCC is the most influential database and provides comprehensive information pertaining to the needs of bibliometric software. In this study, we performed an in-depth literature search using the WoSCC.

Search Strategy

We employed the WoSCC database to gather all pertinent publications. This comprehensive database spans a wide range of scientific literature and serves as a primary resource for bibliometric software statistics. Hence, it is the predominant database for bibliometric investigations [5-7]. By June 1, 2025, we downloaded all relevant studies from the WoSCC archive. Our search strategy for identifying articles discussing ACA relied on the following criteria: TOPIC = (TS=(Adenomas, Adrenocortical) OR TS=(Adrenocortical Adenomas) OR TS=(Adenoma, Adrenal Cortical) OR TS=(Adenomas, Adrenal Cortical) OR TS=(Adrenal Cortical Adenoma) OR TS=(Adrenal Cortical Adenomas) OR TS=(Adenoma, Adrenocortical). To maintain consistency and avoid discrepancies, we restricted our search to publications until June 1, 2025.

Data Extraction and Analysis

VOSviewer (1.6.18) serves as a bibliometric analysis software capable of extracting key information from a multitude of publications [8]. In this study, VOSviewer primarily facilitated the execution of several analyses, i.e., co-occurrence analysis across three modules (density, overlay, and network visualization) for institutions, authors, countries, and keywords. As a result, a visual atlas depicting the links between research hotspots and scientific literature in many dimensions was created. A node on the VOSviewer map denotes an item (a nation, organization, publication, or writer), and the size and color of the node indicate the number and kind of objects, respectively. Meanwhile, the line thickness between nodes shows how closely related or co-cited an item is [9,10]. CiteSpace (6.1.R1) was developed by Professor Chen C for bibliometric analysis and visualization [11].

**Figure 1:**
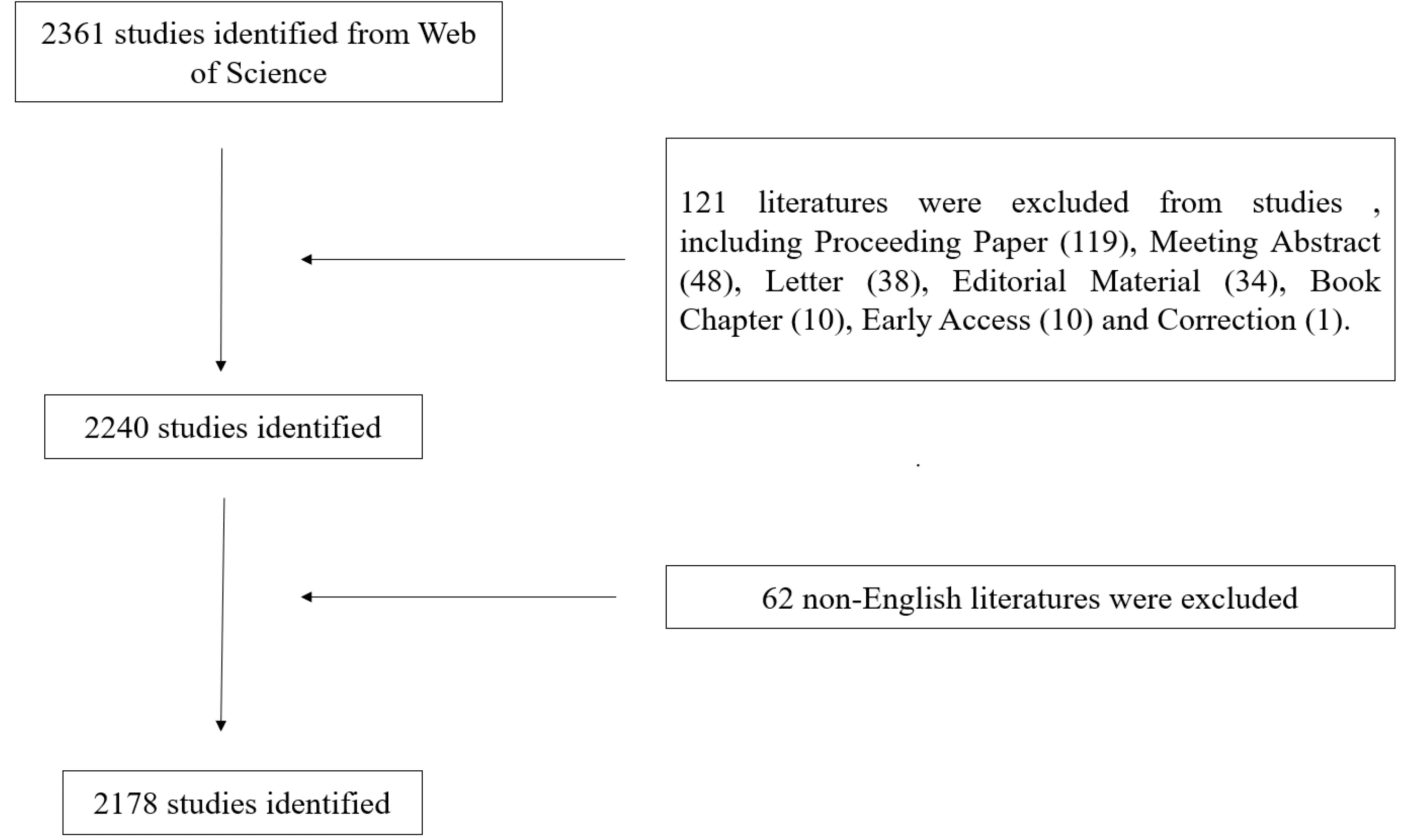
Flowchart showing the comprehensive search procedure.

A total of 2,361 articles related to ACA were retrieved from the WoSCC database. These articles fell into six categories, as presented in Table 1. The most common type was an Article (1,852), accounting for 78.45% of all publications. The second most prevalent category was Review Article (388), which constituted 16.44% of the total. The remaining seven categories included Proceeding Paper (119), Meeting Abstract (48), Letter (38), Editorial Material (34), Book Chapter (10), Early Access (10), and Correction (1). Following the exclusion of 62 non-English-language articles, the final selection for this study comprised original research articles, reviews, and meta-analyses related to the ACA. A total of 2,178 articles were ultimately analyzed, with the detailed filtering process illustrated in Figure 1.

**Table 1:** Publication document types.

Rank	Document types	Counts	% of 2,361	Total citations	H-Index
1	Articles	1,852	78.45	54,178	98
2	Review article	388	16.44	12,949	55
3	Proceeding paper	119	5.04	4,447	37
4	Meeting abstract	48	2.03	0.00	0
5	Letter	38	1.61	174	5
6	Editorial material	34	1.44	155	7
7	Book chapter	10	0.42	169	8
8	Early access	10	0.42	4	1
9	Correction	1	0.04	0	0

Data Analysis and Visualization

Through the integration of citation analysis, co-occurrence analysis, and complex network analysis, the software effectively reveals crucial information, including scientific collaboration networks, research frontier progression, knowledge base frameworks, the spread of research hotspots, and emerging trends in specific fields. This provides researchers with data-driven perspectives on advances in the field.

To this end, we employed CiteSpace (version 6.2.R3) and VOSviewer (version 1.6.19) for collaboration network analysis (encompassing country/region, institution, author, and journal), co-citation analysis (focusing on author, journal, and references), and dual-map overlay of the literature. The precise configurations utilized in CiteSpace were as follows: time slicing set from January 2000 to June 2025, with annual intervals; text processing encompassing title, abstract, and author keywords; node types selected individually from country, institution, author, keyword, co-cited journal, co-cited author, and co-cited reference, with selection criteria based on a g-index of K = 25 or K = 20; and pruning of the sliced networks, all adhering to default settings.

VOSviewer, developed by the Centre for Science and Technology Studies at Leiden University in the Netherlands, is a leading visualization tool for constructing scientific knowledge maps [12,13]. The software is known for its powerful network analysis capability and intuitive visualization effect, which can efficiently process large-scale bibliometric data, realize the visual presentation and deep mining of complex networks, such as scientific collaboration networks, keyword co-occurrence networks, and literature coupling relationships, and is used to create visual charts and analyze the most active/collaborating countries, institutions, and authors, as well as the most frequently cited journals and the most frequent keywords. Keywords. This helps create a visual atlas that illustrates research hotspots and connections between scientific literature across dimensions. In VOSviewer, each node represents an entity, and the size and color of the nodes reflect the number and type of entities, respectively. In addition, the thickness of the lines between the nodes indicates the degree of association or co-citation of the entities [14,15].

Results

Temporal Distribution Map of Publications and Citations

**Figure 2:**
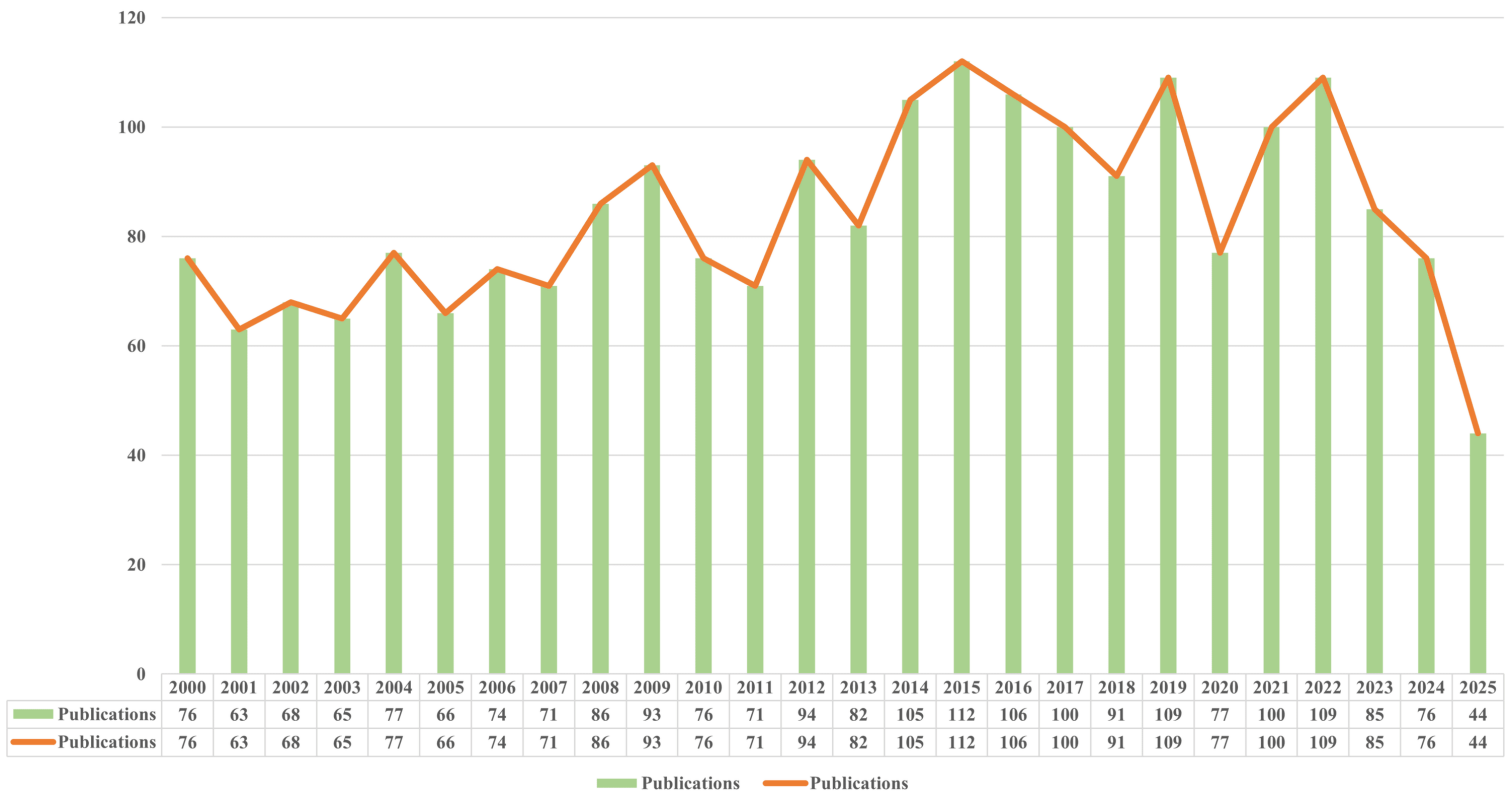
Annual publication volume trends from 2000 to 2025.

Changes in annual publications and citation frequency reflect the pace and progress of research as well as the degree of research focus on this topic [16,17]. From 2000 to 2025, annual publications on ACA aspects included 2,178 relevant articles, showing a steady overall trend (Figure 2).

The number of articles published in the early part of the decade (2000-2010) fluctuated between 60 and 95 articles, with no apparent sustained growth or downward trend. The highest number of publications was in 2009 (n = 93), and the lowest was in 2001 (n = 63). The period from 2011 to 2015 showed rapid growth, when the number of articles entered a clear upward trajectory, maintaining growth or high levels in all years except 2013 (n = 82), when there was a slight pullback, and peaking in 2015 (n = 112), which was a nearly 50% increase in the number of articles over this five-year period. The period from 2016 to 2021 was a period of turbulence and fluctuation, with a large decline in 2018 (n = 91), followed by a strong rebound in 2019 (n = 109), almost reaching the peak level before 2020 (n = 77) turned to a sharp decline and a significant rebound in 2021 (n = 100). The period from 2022 to 2025 was a period of continuous decline. It is worth noting that the data collected for this article ends on July 1, 2025, so the number of publications (n = 44) is only a provisional reference and has not yet been fully counted; the actual number of publications for the whole year may be significantly higher than the current data.

Analysis of Leading Countries, Regions, and Institutions

**Figure 3:**
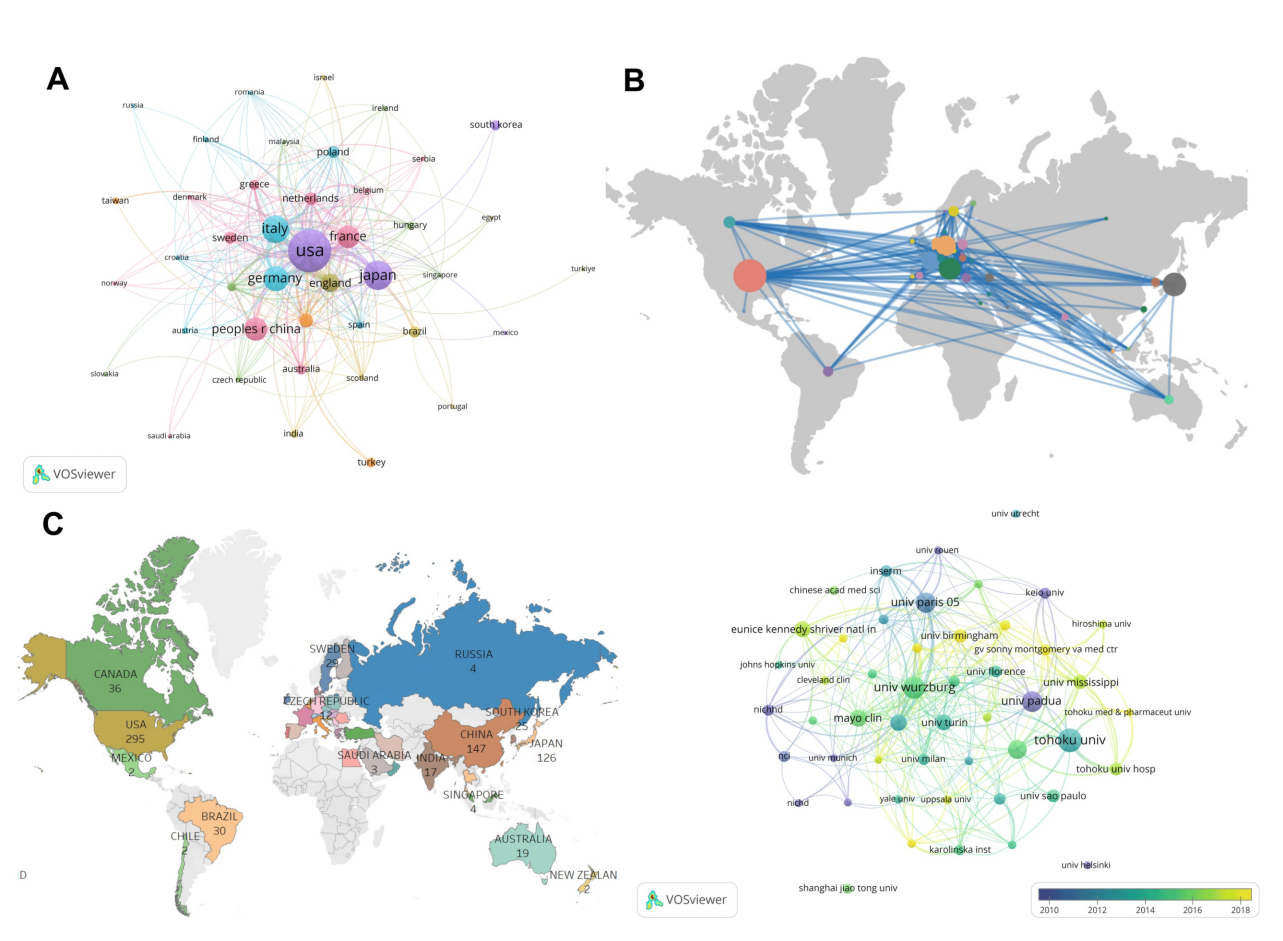
(A) Visualization of countries. (B) Country/Region cooperation map on adrenal cortical adenoma. (C) Network map of intercountry/interregional cooperation. (D) Network map of institutions.

The top 10 contributing countries and regions were identified by analyzing a map of collaborative networks in ACA-related studies (Table 2, Figure 3, Panel A). We screened 42 countries for inclusion in the study based on countries with five or more publications. The heat world map (Figure 3, Panel B) shows that these articles are mainly concentrated in North America, East Asia, and Western Europe. The United States ranked first in the world, with 606 postings. This was followed by Japan (311), Italy (270), Germany (231), and China (200). This distribution not only highlights the all-encompassing nature of the ACA research field but also demonstrates the broad geographic diversity of the field, reflecting the fact that a small number of other countries in South America and Oceania are also actively involved in the field. The size of each circle in the network map represents the number of papers published in the respective country, and the thickness of the connecting lines indicates the density and frequency of collaboration between countries and regions.

**Table 2:** The 10 most productive countries and institutions in adrenal cortical adenoma research.

Rank	Country	Counts	Institution	Counts
1	USA (North America)	606	Tohoku University (Japan)	101
2	Japan (Asia)	311	University of Wurzburg (Germany)	86
3	Italy (Europe)	270	University of Padua (Italy)	80
4	Germany (Europe)	231	Paris Descartes University (France)	76
5	China (Asia)	200	University of Michigan (USA)	66
6	France (Europe)	195	Mayo Clinic (USA)	57
7	England (Europe)	131	Hospital Cochin (France)	52
8	Canada (North America)	80	Polytechnic University of Turin (Italy)	49
9	Netherlands (Europe)	68	Eunice Kennedy Shriver National Institute of Child Health and Human Development (USA)	48
10	Sweden (Europe)	62	University of Mississippi (USA)	40

This analysis revealed differences in the extent of ACA research conducted in different regions worldwide. It is worth noting that overall, a relatively close and extensive network of collaboration has been formed in ACA research globally (Figure 3, Panel C). The United States, Australia, Canada, Brazil, and Japan have closer collaborations, indicating a growing trend of cross-border and international research collaborations in the field. In addition, we also selected institutions with 15 or more publications, resulting in a total of 49 institutions included in the study.

Table 2 shows that the highest number of publications was from Tohoku University (101), followed by the University of Würzburg (86) and the University of Padua (80). Most of the top 10 institutions are located in North America and European countries. On examining the strength of collaborative connections and the institutional network collaboration graph (Figure 3, Panel D), Tohoku University, University of Wurzburg, University of Padua, and Paris Descartes University showed more connectivity, indicating that these institutions collaborated more frequently with each other. Clearly, there is room for enhancing global cross-institutional writing in ACA research.

Analysis of Leading Journals

To search for the most productive and influential journals, we used the VOSviewer software to visualize published journals related to ACA and selected journals with a number of publications greater than or equal to 10. A total of 44 journals were included in the analysis. The results (Table 3) show that 554 academic journals published relevant papers.

**Table 3:** The top 10 productive and co-cited journals in adrenal cortical adenoma research.

Rank	Sources	Counts	JCR	IF (2024)	Co-sources	Citations	JCR	IF (2024)
1	J Clin Endocr Metab	160	2	5.1	J Clin Endocr Metab	10,617	2	5.1
2	Eur J Endocrinol	84	2	5.2	Eur J Endocrinol	3,173	2	5.2
3	Clin Endocrinol	51	3	2.4	Clin Endocrinol	2,119	3	2.4
4	Front Endocrinol	46	2	4.6	New Engl J Med	1,737	1	78.5
5	Endocr J	41	4	2.1	Am J Roentgenol	1,651	2	6.1
6	Horm Metab Res	41	3	1.8	Endocrinology	1,650	3	3.3
7	Endocr Pathol	39	1	14.7	Radiology	1,552	1	15.2
8	J Endocrinol Invest	38	2	3.5	Hypertension	1,396	1	8.2
9	Mol Cell Endocrinol	38	2	3.6	Am J Surg Pathol	1,370	1	4.2
10	Endocr-Relat Cancer	37	2	4.6	Mol Cell Endocrinol	1,332	2	3.6

**Figure 4:**
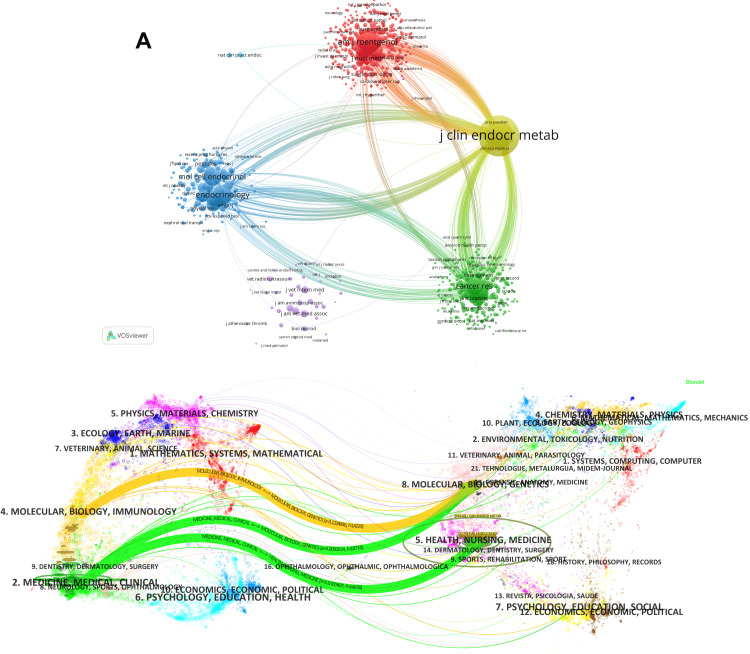
(A) Co-citation network map of journals. (B) Dual-map overlay of adrenal cortical adenoma research.

Among them, J Clin Endocr Metab (160), Eur J Endocrinol (84), and Clin Endocrinol (51) were the top three journals that published research in this field. Moreover, the impact factor (IF) of a journal is an important parameter for assessing its value and that of its publications [14]. Of note, Endocr Pathol has the highest impact factor (14.7), followed by Eur J Endocrinol (5.2). In addition, we selected journals with co-citation greater than 25 and screened 436 journals for inclusion in the study. The analysis of journal co-citations shows each journal’s contribution to the field. The journal co-citation network diagram is presented in Figure 4, Panel A. Among the 5,219 journals co-cited, J Clin Endocr Metab had the highest number of co-citations (10,617, IF = 5.1), followed by Eur J Endocrinol (3,173, IF = 5.2) and Clin Endocrinol (2,119, IF = 2.4).

Among them, 80% of the journals belonged to Q1, Q2 JCR division, and among the 10 most co-cited journals, two journals had an IF of more than 10, among which New Engl J Med (1,737, IF = 78.5) was the most highly co-cited journal with the highest impact factor. The journal has published a review on the management of incidentaloma, covering the differential diagnosis of adrenocortical adenomas and the importance of hormonal assessment [18], as well as an article on adrenal Cushing’s syndrome, which focuses on the role of constitutive activation of protein kinase A catalytic subunit in the pathogenesis of adrenal Cushing's syndrome, revealing how aberrant activation of the protein kinase A signaling pathway leads to hyperproliferation of adrenocortical cells and overproduction of cortisol, thereby triggering Cushing’s syndrome [19]. There are many other notable articles published in the journal, making it one of the most influential clinical medical journals in the world, playing a leading role in renal cell carcinoma research and driving the establishment and innovation of a series of key diagnostic and therapeutic standards.

The essence of the dual graph overlay is the connection between the applicator and cited domains, and the overlay graph can reflect the knowledge flow between disciplines at the journal level [20]. It shows the citation relationship between journals and co-cited journals, with citation clusters on the left, citation clusters on the right, and citation links on the curves, which show the full context of citations [21]. We merged the maps through “Overlay Maps” in Citespace, and after obtaining the preliminary results of the analysis, we merged the curves through the Z-score function (Figure 4, Panel B). It can be seen that the citations related to ACA are mainly related to journals of Medicine, Medical, and Clinical, and there is a significant crossover with Molecular, Biology, and Genetics, illustrating the interdisciplinary nature of ACA research.

Analysis of Leading Authors and Co-cited Authors

**Figure 5:**
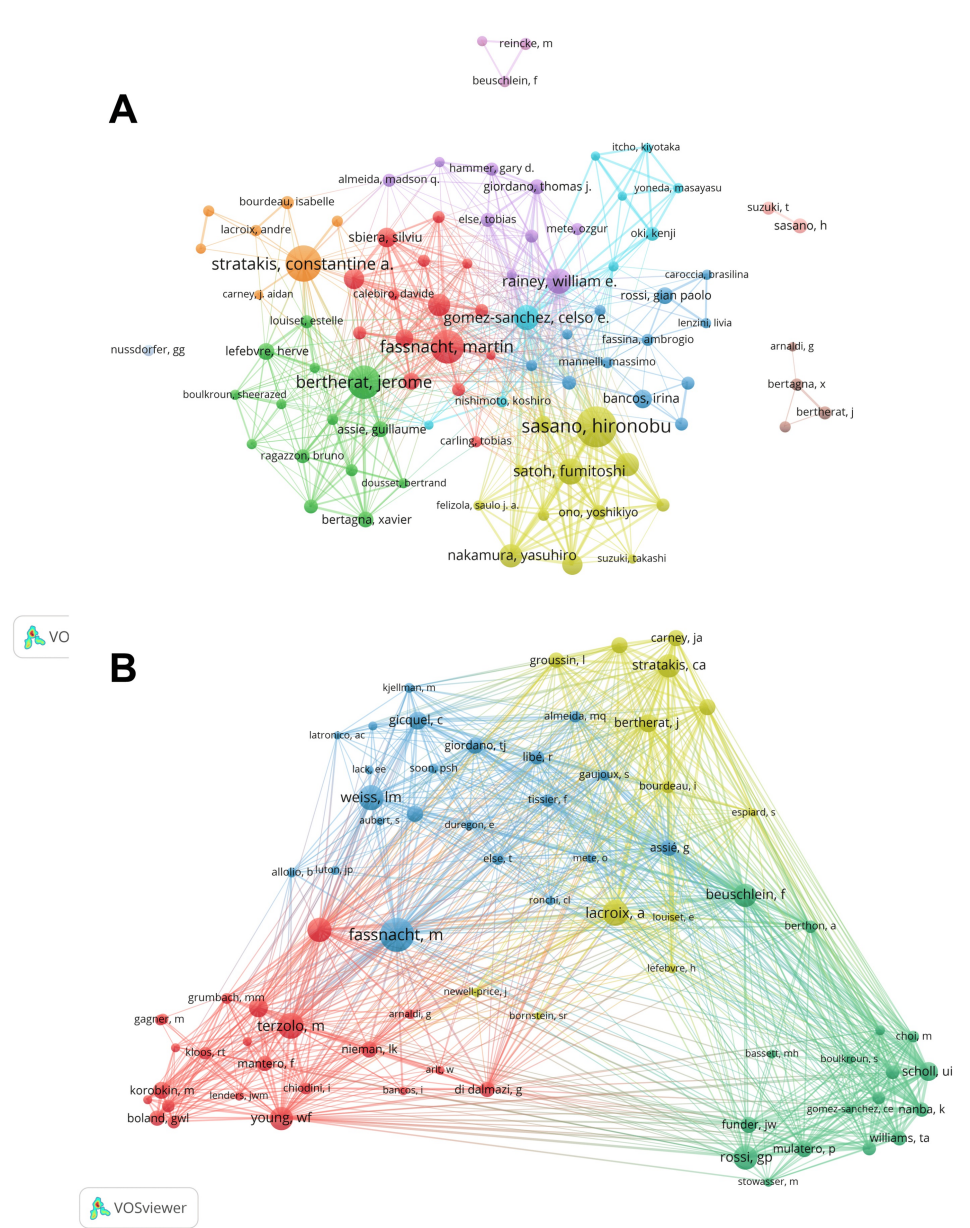
(A) Cooperation map of authors. (B) Co-citation collaboration network of authors.

In total, 10,350 authors were involved in the analyzed publications. In the co-occurrence diagram of the authors’ collaborative network, different clusters are represented by different colored circles, and the lines indicate collaboration intensity. This analysis revealed the existence of 10 major author collaborative clusters, indicating substantial collaboration among authors. We built a collaborative network based on authors whose number of published papers was greater than or equal to 10; ultimately, 89 authors were included in the analysis (Figure 5, Panel A). Sasano Hironobu, Stratakis Constantine, Bertherat Jerome, and Fassnacht Martin have the largest nodes because they published the most related publications. In addition, we observed close collaboration among multiple authors. For example, Sasano Hironobu has close cooperation with Satoh Fumitoshi, Nakamura Yasuhiro, and Ono Yoshikiyo. Bertherat Jerome has active cooperation with Assie Guillaume, among others. Table 4 shows the top 10 authors in terms of the number of publications, with Sasano Hironobu having the largest nodes because he published the most related publications. Sasano Hironobu has the highest number of papers in this field (71), followed by Stratakis Constantine (59), and Bertherat Jerome (54).

**Table 4:** The top 10 most productive authors in adrenal cortical adenoma research from 2000 to 2025.

Rank	Authors	Counts	Co-cited authors	Citations
1	Sasano Hironobu	71	Fassnacht M	635
2	Stratakis Constantine	59	Lacroix A	451
3	Bertherat Jerome	54	Terzolo M	440
4	Fassnacht Martin	53	Weiss LM	428
5	Satoh Fumitoshi	38	Rossi GP	407
6	Gomez-Sanchez Celso	37	Reincke M	400
7	Rainey William	37	Beuschlein F	398
8	Yamazaki Yuto	33	Stratakis CA	389
9	Nakamura Yasuhiro	33	Young WF	387
10	Reincke Martin	31	Scholl UI	289

We also set the number of authors with at least 100 citations, and 75 authors were selected for inclusion in the study. Figure 5, Panel B shows that the top five authors with the highest number of citations are Fassnacht M (635), Lacroix A (451), Terzolo M (440), Weiss LM (428), and Rossi GP (407). There are also active collaborations among different co-cited authors, such as Terzolo M, Fassnacht M, Weiss LM, and Stratakis CA, who have had a significant scholarly impact on the field of ACA.

Analysis of Keywords and Burst Words

Keywords are standardized language chosen from headings and texts to denote the topic of a paper and make it easier to archive information [22]. Keywords can be used to accurately identify research frontiers and hot areas when investigating the knowledge structure of science, which is a useful method for bibliometric analyses. Keyword co-occurrence analysis is a common method for identifying popular research topics. In addition to search terms, author keywords extracted from the titles and abstracts of 2,178 papers were analyzed using VOSviewer. A total of 6,019 keywords were extracted. We retained only the keywords with 50 occurrences, and 70 keywords were included in the study. The top 10 most frequently used keywords were “tumors,” “expression,” “adrenocortical carcinoma,” “diagnosis,” “management,” “carcinoma,” “pheochromocytoma,” “adenoma,” “Cushing’s syndrome,” and “adrenocortical adenoma.” The research hotspots cover the fields of cancer, gene expression, and adrenocortical carcinoma, among others (Table 5).

**Table 5:** The top 20 keywords in adrenal cortical adenoma research.

Rank	Keyword	Occurrences	Rank	Keyword	Occurrences
1	Tumors	326	11	Adenomas	171
2	Expression	320	12	Cancer	170
3	Adrenocortical carcinoma	306	13	Primary aldosteronism	169
4	Diagnosis	300	14	Cushing’s syndrome	160
5	Management	248	15	Adrenocortical tumors	155
6	Carcinoma	227	16	Masses	155
7	Pheochromocytoma	214	17	Somatic mutations	149
8	Adenoma	194	18	Adrenal	127
9	Cushing’s syndrome	193	19	Hypertension	124
10	Adrenocortical adenoma	188	20	Prevalence	123

**Figure 6:**
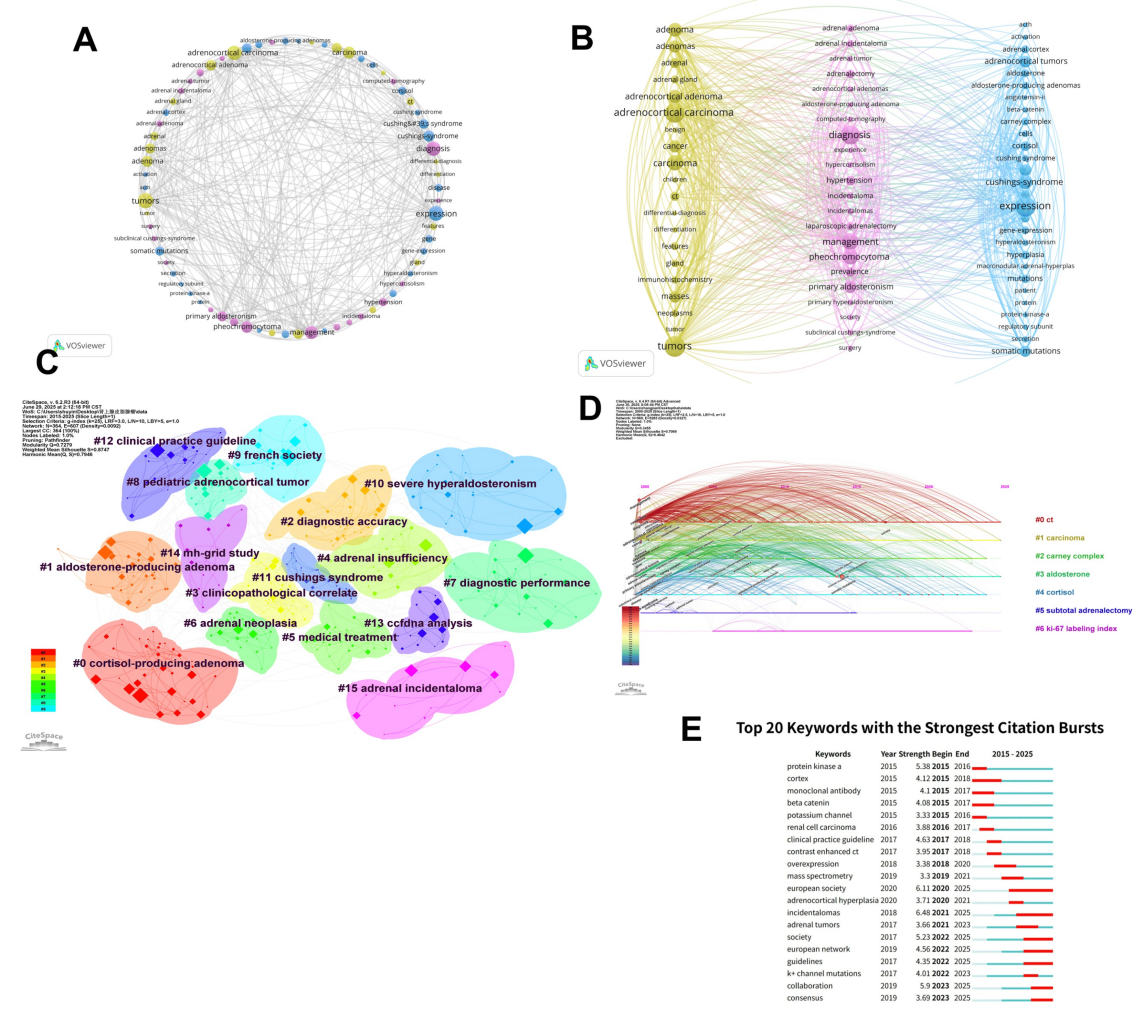
(A) Map of keywords in adrenal cortical adenoma research. (B) Timeline map of keywords in adrenal cortical adenoma research. (C) Cluster view of the keyword clustering analysis. (D) Timeline view of the keyword clustering analysis. (E) Top 20 keywords with major citation bursts.

The network and overlay visualizations of co-occurring keywords are displayed in Figure 6, Panels A and B, showing that all keywords are divided into three clusters, which can be classified into three main thematic categories based on the color distributions of the words in the color columns, with each color representing a specific research direction or field. Among them, the yellow columns on the left side focus on the pathological classification, histological features, and anatomical basis of adrenal tumors, including “adrenocortical carcinoma,” “tumors,” “adrenal gland,” and “differentiation.” The center column, in pink, focuses on diagnostic techniques, clinical symptoms, and treatments for adrenal disease, including “diagnosis,” “Cushing’s syndrome,” “ management,” “differentiation,” and “primary aldosteronism.” The right column, in blue, delves into the molecular mechanisms of adrenal tumors (e.g., gene mutations, protein kinase regulation) and hormonal abnormalities (e.g., aldosterone, cortisol), including “expression,” “cortisol,” “gene expression,” and “somatic mutations.”

The protrusions were clustered into 15 major clusters (Figure 6, Panel C), including “#0 cortisol-producing adenoma,” “#1 aldosterone-producing adenoma,” “#2 diagnostic accuracy,” “#3 clinicopathological correlate,” “#4 adrenal insufficiency,” “#4 adrenal insufficiency,” and “#5 medical treatment,” among others. Each cluster is color-coded to represent a different research area, facilitating the identification of key trends and foci within the study [23].

In addition, we used CiteSpace to visualize the evolution of keywords over time (Figure 6, Panel D). This figure has a Q-value of 0.3455 and an S-value of 0.7069. The timeline of co-cited references visualizes topic distribution over time. The timeline incorporates clustering and time-slicing techniques to illustrate the evolution of research themes. Nodes on the timeline are color-coded to represent different years; nodes on the left are older, and nodes on the right are newer. The upper horizontal axis is the timeline (2000-2025), and the nodes below the timeline indicate the first or active occurrence of the keyword at that point in time. Among them, #0 ct clustering has lasted since 2002, which is the largest and earliest research hotspot, and the keywords are mostly related to imaging, diagnosis, management, and metastasis; #1 carcinoma has appeared since 2005, which focuses on malignant tumors, and is closely related to treatment and malignancy; #2 carney complex has been active since 2007, and this clustering reflects the increased interest in genetic mechanisms in recent years; #3 aldosterone has been active since 2010 and focuses on the link between primary aldosteronism and hypertension, suggesting a rise in the weight of endocrinology in the study of adrenal disorders; #4 cortisol focuses on cortisol secretion and Cushing’s syndrome, shedding further light on the role of hormonal disruption in adrenal disease; and #5 subtotal adrenalectomy and #6 ki-67 labeling index appeared later, focusing on the surgical approach of preserving part of the adrenal tissue and the discovery of Ki-67 as an important marker of tumor proliferation, respectively, suggesting that the application of molecular indexes is becoming more and more important in pathological diagnosis and prognostic assessment.

Figure 6, Panel E shows the top 20 keywords that exhibited the strongest citation bursts between 2000 and 2025. In this visual analysis, the red bars represent terms that have gained a lot of academic attention and frequency of citations over the specified period; conversely, the light green bars represent terms that have decreased in popularity or frequency of citations, indicating a decline in academic attention or relevance over the corresponding period [24]. As can be seen, “European society” became the most cited keyword, and over time, “European network,” “guidelines,” “collaboration,” and other terms may become hot research topics in the coming years.

Co-cited References and References With Citation Bursts

In 1973, Small and Marshakova proposed co-citation analysis as a research tool for assessing links between articles and subsequently integrated it into literature co-citation analysis [25]. When two or more articles appear simultaneously in the reference list of one or more subsequent publications, it is known as a co-citation relationship [15,26,27]. Over the past 20 years, 42,010 publications related to ACA research have been co-cited.

**Figure 7:**
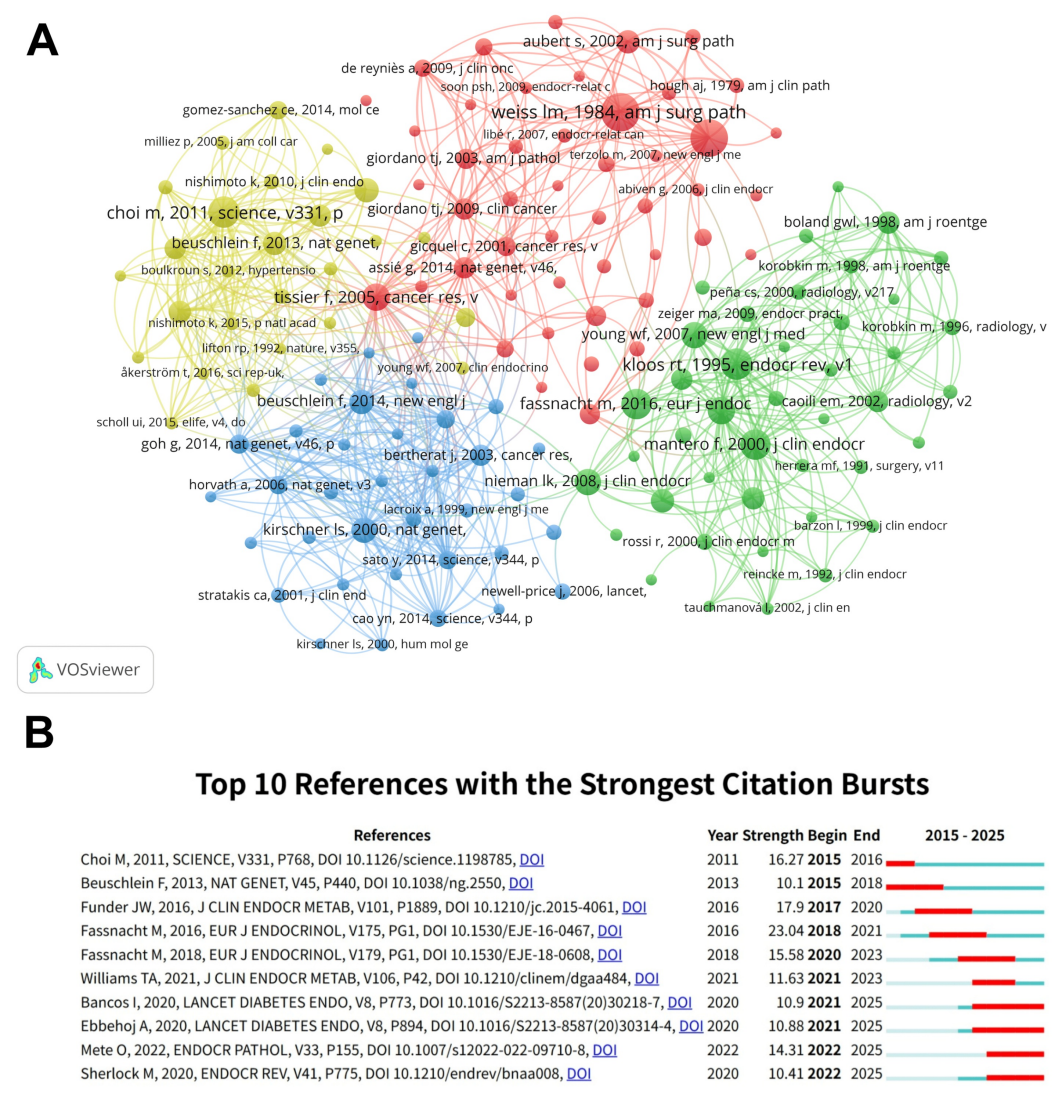
(A) Visualization of co-cited references on adrenal cortical adenoma research. (B) Visual analysis of reference bursts. The intensity reflects the frequency of citations. The red bars indicate citation frequency, and the green bars indicate fewer citations.

In the screening process, we selected literature with more than 40 citations, and 150 articles were included in our analysis. The top 10 co-cited articles listed in Table 6 received at least 130 co-citations. Among them, Weiss et al.’s article “Comparative histologic study of 43 metastasizing and nonmetastasizing adrenocortical tumors” [28] was the most cited (n = 216), followed by “Pathologic features of prognostic significance in adrenocortical Pathologic features of prognostic significance in adrenocortical significance in adrenocortical carcinoma” [29], also published by Weiss et al. in the same journal in 1989, ranking second (n = 207). Using VOSviewer for co-citation analysis, Figure 7, Panel A shows that “Weiss LM, 1984, Am J Surg Path” shows active co-cited relationships with “Beuschlein F, 2014, New Engl J,” “Choi M, 2011, Science, v331, p,” and “Kloos RT, 1995, Endocr Rev, v1,” among others.

**Table 6:** The top 10 co-cited references.

Rank	Co-cited references	Citations
1	Weiss LM, 1984, Am J Surg Pathol, v8, p163	216
2	Weiss LM, 1984, Am J Surg Pathol, v13, p202	207
3	Kloos RT, 1995, Endocr Rev, v16, p460	160
4	Choi M, 2011, Science, v331, p768	160
5	Fassnacht M, 2016, Eur J Endocrinol, v175, pg1	158
6	Mantero F, 2000, J Clin Endocr Metab, v85, p637	156
7	Grumbach MM, 2003, Ann Intern Med, v138, p424	136
8	Tissier F, 2005, Cancer Res, v65, p7622	135
9	Young WF, 2007, New Engl J Med, v356, p601	132
10	Nieman LK, 2008, J Clin Endocr Metab, v93, p1526	132

Citation explosion references are those that have seen a significant increase in the number of citations over a specific period, indicating heightened scholarly interest in a particular topic [30-32]. In our study, Citespace identified 10 references with strong citation bursts, as shown in Figure 7, Panel B, where each bar corresponds to one year, and the brightness period of the red bar is characterized by a significant surge in citations [33]. Citation bursts for references appeared as early as 2011 and as late as 2022. The strongest citation burst (strength = 23.04) was for the article titled “Management of adrenal incidentalomas: European Society of Endocrinology Clinical Practice Guideline in collaboration with the European Network for the Study of Adrenal Tumors” [34], authored by Fassnacht et al., with citation bursts from 2018 to 2021. It is an authoritative clinical practice guideline published by the European Society of Endocrinology in collaboration with the European Network for the Study of Adrenal Tumors [28], with the core focus of providing a standardized, evidence-based process for physicians worldwide for the management of adrenal incidentalomas. Before the publication of the guidelines, the management of adrenal incidentalomas varied widely across the globe and lacked uniform standards; therefore, the guidelines established a clear, uniform pathway of care based on the best available scientific evidence. The guidelines provided physicians with clear recommendations for assessing the malignant potential and hormonal activity of tumors, avoiding over- and under-medication, and preventing unnecessary testing and surgery for the vast majority of benign non-functioning adenomas. This guideline has become the de facto standard and authoritative reference for the management of adrenal incidentalomas worldwide, enhancing the science and safety of diagnosis and treatment. It provides a clear, easy-to-follow roadmap for physicians in endocrinology, urology, general surgery, and imaging, and systematizes and standardizes fragmented knowledge, profoundly changing the way physicians around the globe approach a common clinical problem.

Additionally, the reference with the second strongest citation burst (strength = 16.27) was titled “K+ Channel Mutations in Adrenal Aldosterone-Producing Adenomas and Hereditary Hypertension” [35], authored by Choi et al., with citation bursts from 2015 to 2016. This groundbreaking study, published in Science in 2011, revealed a novel pathogenic mechanism: mutations in the *KCNJ5* gene lead to aberrant potassium channel function, which causes persistent depolarization of adrenal cortical cell membranes, activates inward calcium flow, and, ultimately, leads to uncontrolled over-synthesis and secretion of aldosterone, leading to hypertension and hyperkaliemia. At that time, the underlying cause of the majority of non-hereditary APA was unknown, and researchers hoped that modern gene sequencing techniques would lead to the identification of “driver” mutations that cause the development of these benign tumors and the overproduction of hormones, as well as the investigation of the presence of genetic defects similar to those in APAs, which could explain the development of some rare but severe familial adenomas. Choi et al. identified “hotspot” somatic mutations in the *KCNJ5* gene in the 22 APA tumor samples studied by whole-exome sequencing and other methods, implying that these mutations occur later in tumor cells and are not inherited.

They demonstrated that these mutations disrupt the ion selectivity of potassium ion channels and that the mutated channels abnormally allow a large influx of sodium ions into the cell, leading to a sustained depolarization of the cell membrane and a subsequent sustained increase in the intracellular concentration of calcium ions, which, in adrenocortical cells, is a key signal for initiating the synthesis and secretion of aldosterone. Thus, this mutation ultimately leads to an autonomous, sustained overproduction of aldosterone.

The publication of this article was a watershed event in the field of aldosterone and hypertension research, as it unraveled the “black box” of primary aldosteronism, unifying the etiology of both non-hereditary and hereditary diseases. The *KCNJ5* mutation assay has become an important tool in clinical and research settings, making the development of drugs that specifically target mutated *KCNJ5* channels a promising therapeutic strategy. Although no drugs are currently on the market, this discovery points to a clear direction for future drug development. Overall, this groundbreaking research not only solves a long-standing medical problem but also opens up entirely new avenues for research, diagnosis, and future treatments in the field. This classic text must be read by all endocrinologists and hypertension researchers.

Discussion

The bibliometric analysis presented in this study provides an insightful exploration of research trends, collaborations, and knowledge evolution surrounding ACA. Over the past two decades, ACA research has experienced steady growth, particularly from 2011 to 2015, when the number of publications sharply increased. This reflects an enhanced focus on ACA, likely due to advances in molecular biology, imaging technologies, and diagnostic techniques. Additionally, the decline in publications in the last few years could be attributed to the culmination of foundational studies, which may have led to fewer new discoveries in this well-established field. However, it is anticipated that 2025 will bring more publications as the field continues to progress and mature.

The results of the analysis of leading countries and institutions reveal widespread international interest in ACA, with the United States, Japan, and Italy emerging as the top contributors. The strong collaborative networks between countries, such as the United States, Australia, Canada, and Japan, coupled with the substantial output of institutions such as Tohoku University, suggest that ACA research is becoming an increasingly global endeavor. The geographic distribution of research reflects the interdisciplinary nature of the field, with significant contributions from endocrinology, pathology, genetics, and molecular biology. Collaboration networks in ACA research also show a marked increase in inter-country collaborations, which bodes well for further advancements in ACA understanding.

The analysis of leading journals and co-cited journals points to the dominance of clinical and endocrinology journals, notably J Clin Endocr Metab and Eur J Endocrinol, which have consistently published the most ACA-related research. The high IFs of these journals indicate their authoritative role in disseminating significant findings in ACA research. Furthermore, the co-citation analysis underlined the substantial interconnections between prominent journals, such as the New Engl J Med, which published pioneering research on the management of adrenal incidentalomas and adrenal Cushing’s syndrome, contributing significantly to the advancement of ACA research.

Author collaboration maps reveal key contributors such as Sasano Hironobu, Stratakis Constantine, Bertherat Jerome, and Fassnacht Martin, who have not only led in terms of publications but also played a pivotal role in fostering academic partnerships across multiple research centers. The clustering of these authors into specific research domains highlights their expertise in different aspects of ACA, from genetic mechanisms and molecular profiling to diagnostic and therapeutic strategies.

Innovations in therapeutics for ACA are expected to focus on targeted therapy. With an increasing understanding of the genetic and molecular basis of the disease, drugs targeting specific pathways are being developed. For example, mutations in *PRKACA* have been identified in cortisol-producing adenomas, and targeting the PKA pathway may lead to new therapeutic options [36,37]. Predictive modeling and personalized medicine are emerging as important areas of research in ACA. The construction of prognostic models can help predict patient outcomes and guide treatment decisions. For example, a model using age at diagnosis and saline infusion test parameters can predict APA from idiopathic adrenal hyperplasia with a certain sensitivity and specificity [38]. Nomograms incorporating clinicopathological predictors have been developed to predict overall survival in adult patients with adrenocortical carcinoma after surgery, showing good calibration and discrimination [39].

Genomic research is likely to significantly impact ACA management. Integrated genome-wide analysis has identified novel molecular pathways associated with adrenocortical carcinoma, such as oncostatin M signaling, which may be a druggable pathway [40].

Pharmacological management is used in some cases of ACA. Metyrapone, a steroidogenesis inhibitor, has been shown to be effective in controlling cortisol excess in patients with Cushing’s syndrome. In a retrospective multicenter study of 195 patients with Cushing’s syndrome, including those with adrenal adenoma, metyrapone monotherapy led to significant improvements in biochemical parameters, such as mean serum cortisol day-curve, 9 am serum cortisol, and 24-hour urinary free cortisol [41]. Surgical intervention is a common treatment for ACA. Laparoscopic adrenalectomy is the gold standard for benign adrenal tumors. In a study of 50 patients who underwent laparoscopic adrenalectomy for adrenal lesions ≥6 cm, the final histopathological diagnoses in the majority of patients were adenoma and pheochromocytoma, and no local or distant recurrences were found during the five-year follow-up [42].

One of the most compelling findings of this study is the identification of emerging keywords and burst terms. The frequent appearance of terms such as “aldosterone-producing adenoma,” “carcinoma,” and “Cushing’s syndrome” illustrates the growing recognition of the pathological, genetic, and hormonal mechanisms underlying ACA. The research clusters analyzed in this study revealed a growing interest in genetic mutations, particularly the identification of mutations in the *KCNJ5* gene, which has revolutionized the understanding of APA and hypertension. This discovery has opened new therapeutic avenues, and the *KCNJ5* mutation assay has become an important tool in clinical and research settings. Citation bursts in this area further emphasize its significance in advancing the field.

Additionally, the burst of citations related to clinical practice guidelines for the management of adrenal incidentalomas further emphasizes the importance of standardized approaches for the diagnosis and treatment of ACA. The European Society of Endocrinology guidelines have become a de facto standard for managing adrenal incidentalomas, highlighting the translation of scientific research into practical clinical applications.

## Conclusions

This bibliometric profile of 2,178 ACA publications (2000-2025) showed a surge in output during 2011-2015; dominant contributions from the United States, Japan, and Italy; and leading roles for Tohoku University and the University of Würzburg. Co-citation and keyword bursts highlighted three converging themes, namely, histopathological classification, clinical diagnosis and treatment, and molecular mechanisms. The 2016 European Society of Endocrinology guideline on adrenal incidentalomas remains the strongest citation burst, underscoring its global impact on evidence-based management of adrenal incidentalomas.
